# Pembrolizumab-Associated Stevens-Johnson Syndrome in a Patient With Metastatic Non-small Cell Lung Cancer: A Case Report

**DOI:** 10.7759/cureus.41439

**Published:** 2023-07-06

**Authors:** Michael Sandhu, Binod KC, Jenish Bhandari, Harvir S Gambhir, Ramsay Farah

**Affiliations:** 1 Department of Medicine, Upstate University Hospital, Syracuse, USA; 2 Department of Dermatology, Upstate University Hospital, Syracuse, USA

**Keywords:** stevens-johnson syndrome, cancer immunotherapy, metastatic non-small cell lung cancer, dermato-oncology, pembrolizumab cutaneous side effect

## Abstract

Pembrolizumab is a monoclonal antibody that binds to the programmed cell death-1 (PD-1) receptor and is approved for the treatment of several malignancies. We present a rare case of Stevens-Johnson syndrome (SJS) occurring in a 75-year-old female 14 days after receiving the first dose of pembrolizumab therapy to treat stage IV non-small cell carcinoma of the lungs with metastasis to the brain. Although pruritus and papular, erythematous rashes are documented after its use, severe reactions such as SJS and toxic epidermal necrolysis (TEN) are rarely seen in clinical practice. In addition to supportive care, the patient also received intravenous immunoglobulin (IVIG) and corticosteroid therapy and responded well to the therapy. Nearly complete re-epithelialization was achieved four weeks after the start of skin lesions. This case highlights a rare phenomenon of SJS- and TEN-associated adverse reactions following treatment with pembrolizumab.

## Introduction

Stevens-Johnson syndrome (SJS) and toxic epidermal necrolysis (TEN) are severe cutaneous drug eruptions characterized by extensive necrosis and detachment of the epidermis. Mortality from this can be as high as 35% and most often presents as an adverse drug reaction [1,2]. Medications most commonly associated with SJS/TEN include beta-lactams, nonsteroidal anti-inflammatory drugs (NSAIDs), anticonvulsants, barbiturates, and allopurinol. SJS/TEN is rarely reported with immune checkpoint inhibitors (ICIs) such as pembrolizumab [2]. Our patient presented with SJS only 14 days after receiving the first dose of pembrolizumab therapy for the treatment of metastatic stage IV non-small cell carcinoma of the lungs.

## Case presentation

A 75-year-old Caucasian female with stage IV non-small cell lung cancer complicated by multiple metastatic brain lesions presented to the hospital with a new-onset rash. She received whole-brain radiotherapy for brain metastasis 30 days before the first dose of pembrolizumab (200 mg intravenously {IV}). She did not report any history of recent infection, recent pain medication use, or recent antibiotic use. The patient also had a history of hypertension but was not taking any medication for this, and the patient also had asthma for which she used an albuterol inhaler as needed. She denied any supplement use.

Fourteen days after the first dose of pembrolizumab, she noticed a rash and itching. The rash began over the lower extremities and eventually progressed to the upper abdomen, back, and upper extremities, subsequently blistering over the course of seven days. She then developed significant pain and presented to the oncology clinic where she received intravenous methylprednisolone 60 mg once, followed by prednisone 60 mg daily, and she was advised to continue prednisone until the next office visit. However, her symptoms continued to worsen, and she presented to an outside hospital. She was transferred to our institution for comprehensive dermatology evaluation and further management.

**Figure 1:**
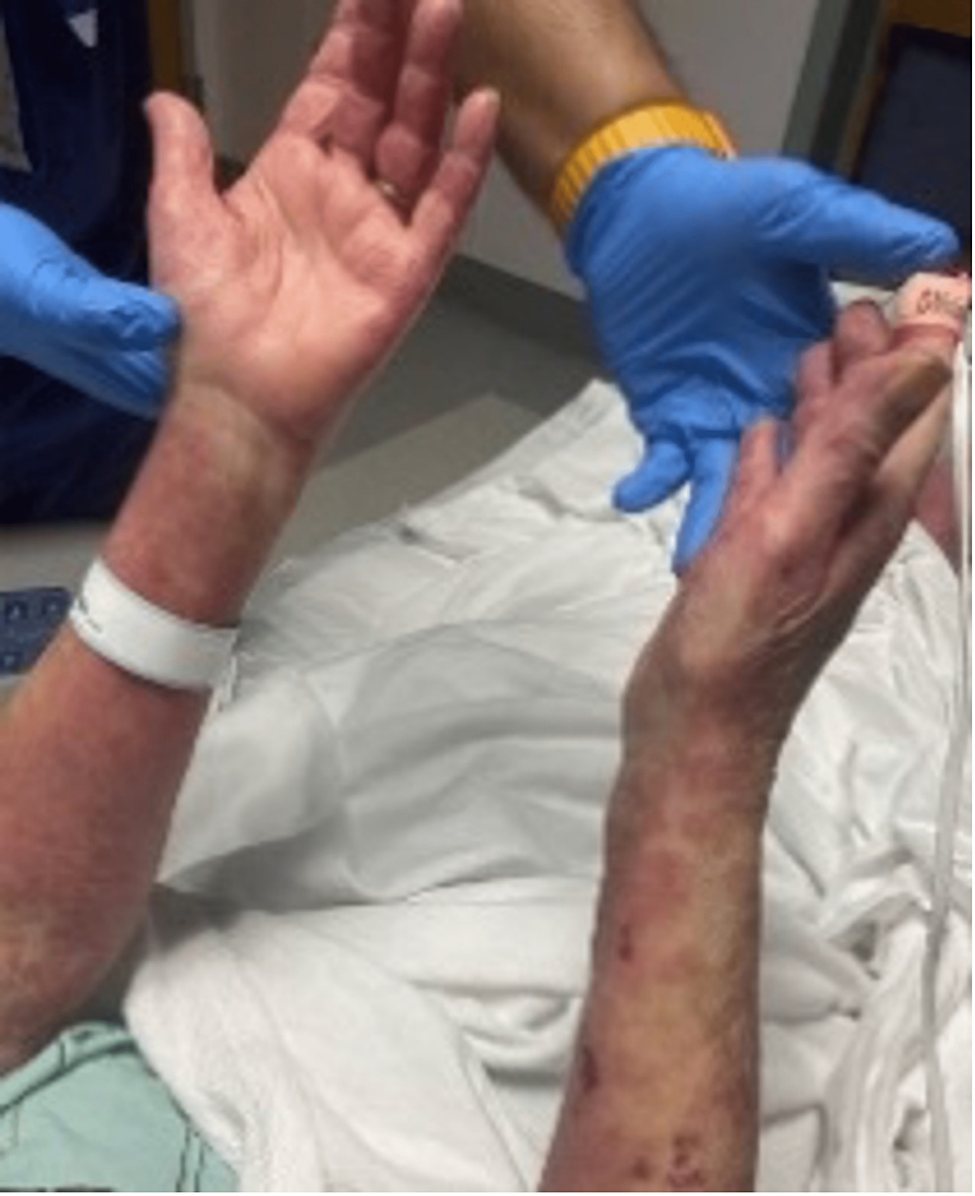
Widespread erythematous cutaneous papules and patches with the detachment of large sheets of necrolytic epidermis over the upper extremities

**Figure 2:**
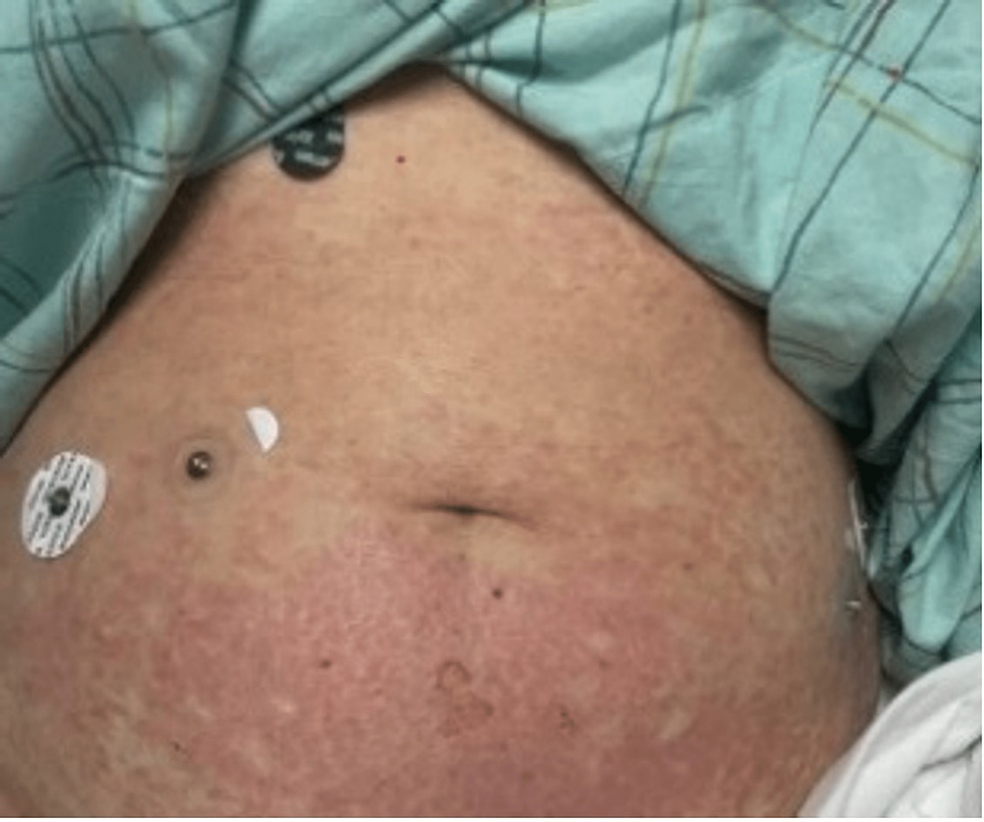
Widespread erythematous cutaneous papules and patches with the detachment of large sheets of necrolytic epidermis over the abdomen

**Figure 3:**
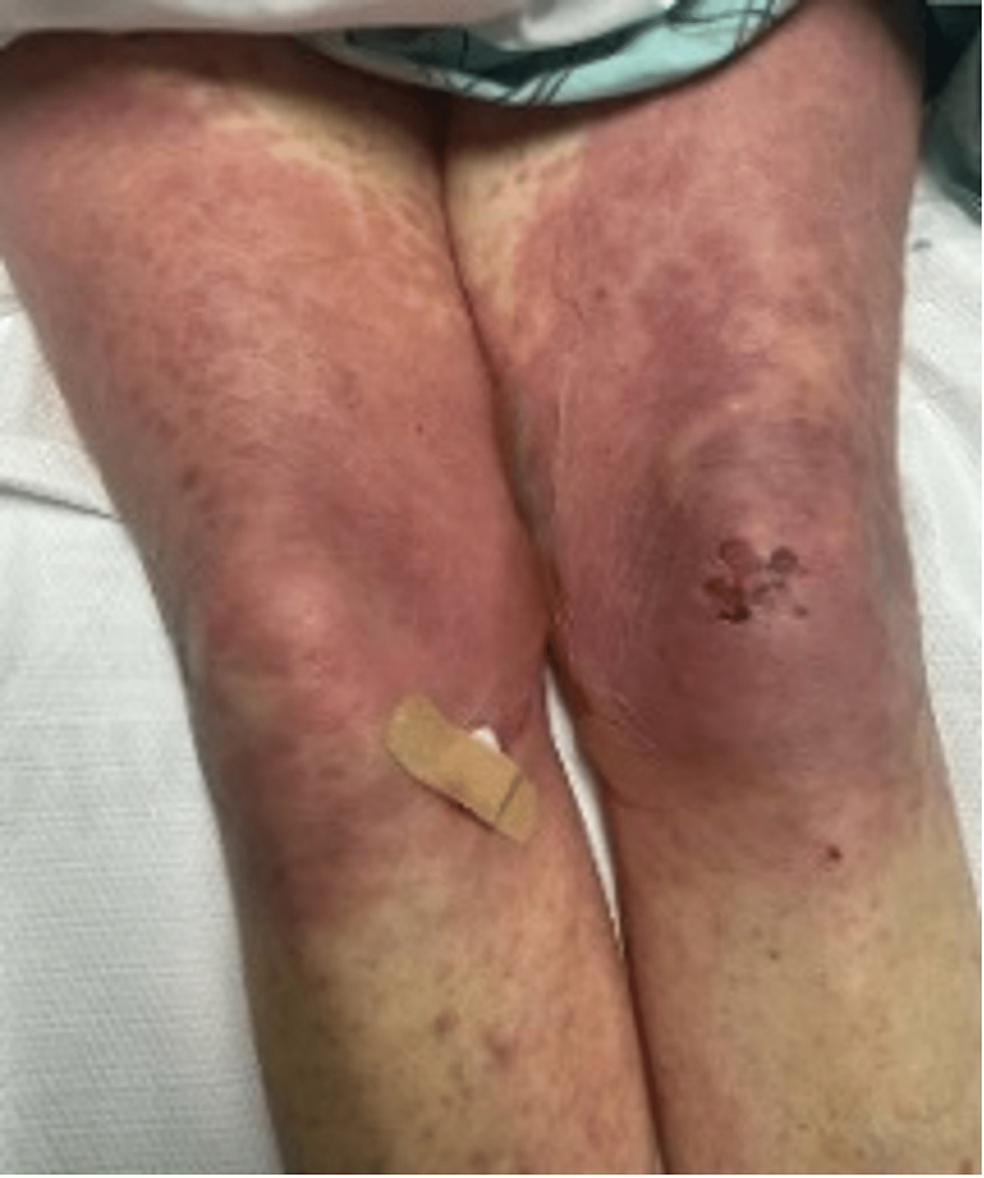
Widespread erythematous cutaneous papules and patches with the detachment of large sheets of necrolytic epidermis over the lower extremities

**Figure 4:**
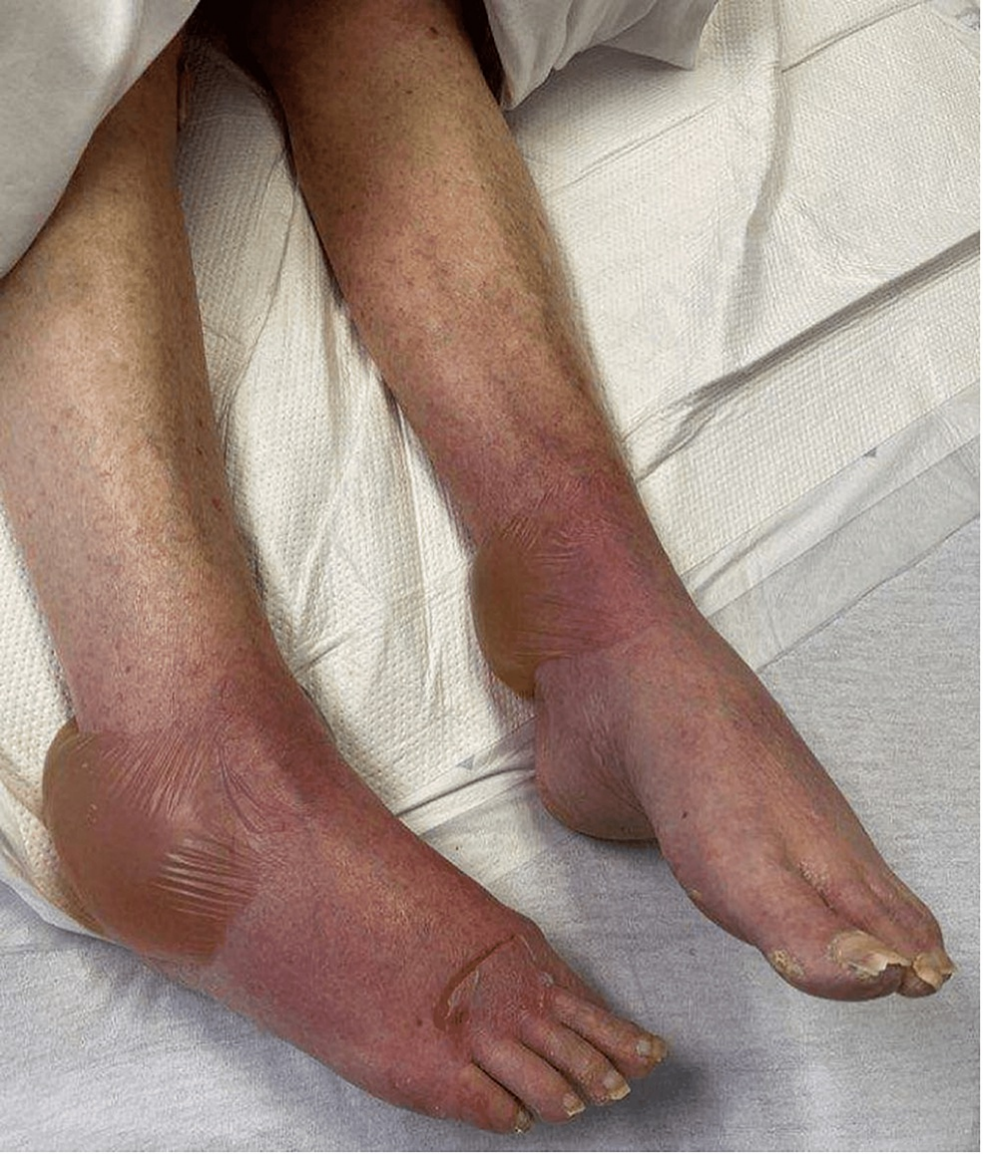
Widespread erythematous cutaneous papules with bullae formation over the lower extremities

Upon presentation, physical examination revealed widespread erythematous papules and papulovesicular changes involving the bilateral upper and lower extremities, as well as the abdomen. She had blistering lesions, most prominent on the feet and thigh. She also had sloughed-off skin on the upper back and bilateral shoulders as demonstrated in Figures 1-4.

**Figure 5:**
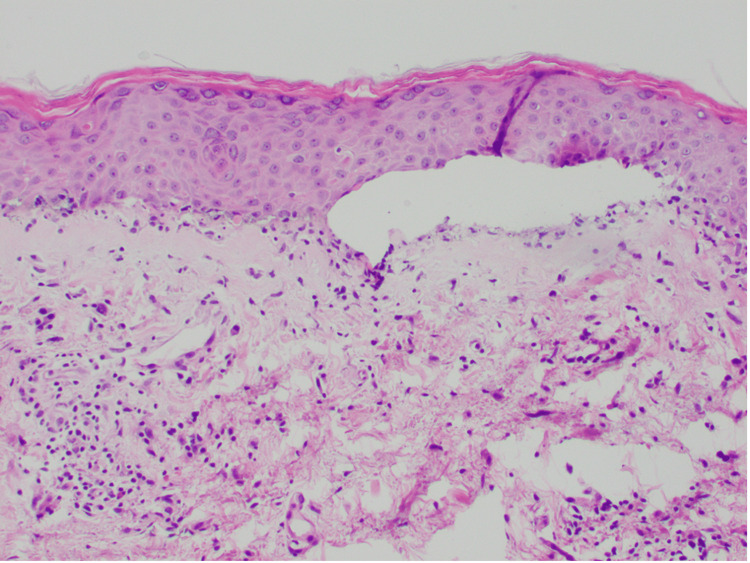
Abdominal skin biopsy showing normal stratum corneum, subepidermal bulla formation with individual cell necrosis, and vacuolar interface dermatitis

The conjunctival congestion of both eyes and the involvement of the oral mucosa were noted. Genital mucosa was not involved. Skin punch biopsies from the abdomen were obtained, which showed normal stratum corneum, vacuolar interface dermatitis, individual cell necrosis, and subepidermal bulla formation (Figure 5), which support the diagnosis of erythema multiforme/SJS/TEN. Due to the patient’s rapid progression, clinical morphology, body surface area (BSA) of <10% involved by bullae or erosions, and pathology consistent with SJS, the decision to initiate treatment for SJS was made. The patient was treated with intravenous immunoglobulin (IVIG) 1 g/kg/day for three days, followed by methylprednisolone IV 60 mg daily for eight days alongside ongoing supportive care. Her methylprednisolone was transitioned to oral prednisone 60 mg and tapered over 30 days. She also received sulfamethoxazole-trimethoprim 800-160 mg oral daily for *Pneumocystis carinii* prophylaxis (PCP) during her hospital stay. At the time of discharge, her rash and symptoms had improved significantly.

Patient perspective

The patient tolerated her therapy well. She had marked improvement in her rash with corticosteroid therapy and has not had further worsening of the rash upon follow-up.

## Discussion

Pembrolizumab is a commonly used humanized monoclonal immunoglobulin G4 (IgG4) antibody approved as a therapy for various malignancies, including non-small cell lung cancer (as used in our patient), Hodgkin lymphoma, late-stage melanoma, and gastric cancer. The mechanism of action of this drug involves the disruption of the interaction between the programmed cell death-1 (PD-1) receptor and programmed death-ligand 1 (PD-L1) [1].

The adverse effects of pembrolizumab vary from commonly seen mild effects such as pruritus and papular, erythematous rash to severe and rarely encountered reactions including SJS/TEN as presented in our patient [3,4]. Clinically, in discussing the spectrum of SJS/TEN, SJS involves 10% of the body surface area (BSA) with erosions or bullae, SJS/TEN overlap involves 10%-30% of the BSA, and TEN involves 30% of the BSA [2]. TEN is a life-threatening drug reaction that usually begins as painful red coalescent erythema and progresses to bulla formation with areas of epidermal detachment with mucosal involvement [2].

The severity of SJS/TEN is defined based on the Severity-of-Illness Score for Toxic Epidermal Necrolysis (SCORTEN). SCORTEN is used to estimate mortality in the patients presenting with SJS/TEN based on the patient’s history, current vital signs, and laboratory values [3]. In our case, our patient’s initial SCORTEN was 3, which was consistent with an estimated mortality of 35.3%.

The interval between drug initiation and SJS/TEN is approximately 5-28 days [5]. Clinical correlation with histopathological findings such as full-thickness epidermal necrolysis, subepidermal bulla formation, individual cell necrosis, and vacuolar interface dermatitis is essential in the diagnosis of SJS/TEN. In our case, the cutaneous eruption started 14 days after the first dose of pembrolizumab. The differential diagnosis based on histology included SJS/TEN, erythema multiforme, severe graft versus host disease, and a bullous fixed drug eruption. The decision to promptly initiate IVIG was made in consultation with dermatology due to the rapid progression and the lack of improvement with outpatient steroid treatment, which was most consistent with SJS. Our patient also exhibited oral mucosal involvement and conjunctival involvement but did not exhibit genital mucosal involvement.

The management of SJS/TEN secondary to immunotherapy is mainly supportive and includes discontinuing the offending drug; initiating short-term pulse-dosed corticosteroids, cyclosporine, and intravenous methylprednisolone at 1-2 mg/kg/day; and close monitoring for acute complications of fluids and electrolyte imbalance, renal failure, bacteremia, hypercatabolic state, insulin resistance, and multiple organ dysfunction syndromes. In severe cases, IVIG can be used. Upon complete re-epithelialization, corticosteroid therapy should be tapered over a minimum of four weeks [2-6].

Riano et al. [7] reported a case of SJS in an 80-year-old female who was treated with six doses of pembrolizumab. In their case, the patient developed skin eruptions and a rash consistent with SJS 15 weeks after the last dose of pembrolizumab [7]. Similar to our case, they reported resolution of the rash after initiation of prednisone, and the patient did not develop any further rash at the one-month follow-up [7]. In another case report by Chow et al. [8], a 63-year-old male with metastatic lung adenocarcinoma presented with TEN approximately 10 weeks after the first cycle of pembrolizumab, carboplatin, and pemetrexed. Due to the significant skin involvement, the patient was treated with high-dose methylprednisolone, cyclosporine, intravenous immunoglobulin, antibiotics, and skin care [8]. They reported excellent recovery of skin lesions with minimal sequelae [8], further supporting the role of immunosuppressive therapy in the treatment of SJS/TEN.

Therefore, we present a rare yet life-threatening complication of SJS seen with the immune checkpoint inhibitor, pembrolizumab, as well as its management with IVIG and long-term corticosteroids.

## Conclusions

SJS and TEN are rare but life-threatening clinical conditions that are most often caused by a reaction to certain drugs. Early identification and management are essential in preventing morbidity and mortality in these patients. Through this case report, we hope to educate clinicians about a rare yet life-threatening complication of SJS seen with the immune checkpoint inhibitor, pembrolizumab, as well as its management with IVIG and long-term corticosteroids.
